# Liver stiffness measured by transient elastography is associated with hepatic Fibrosis in children with portal vein thrombosis: a retrospective cross-sectional study

**DOI:** 10.3389/fped.2026.1779390

**Published:** 2026-06-05

**Authors:** Marwah Imad Shihab, Noor Mohammed Abdullah, Abdulnaser Karem Mhmeed, Enas Osama Hassan Omer, Aya Ahmed Shimal, Ahmed Osama Hassan Omer, Ahmed Dheyaa Al-Obaidi

**Affiliations:** 1 College of Medicine, Al-Iraqia University, Baghdad, Iraq; 2 Faculty of Medicine, Bahri University, Khartoum, Sudan; 3 College of Medicine, University of Baghdad, Baghdad, Iraq; 4 Faculty of Medicine, Al Neelain University, Khartoum, Sudan

**Keywords:** extrahepatic portal vein obstruction (EHPVO), liver stiffness measurement (LSM), pediatric liver fibrosis, portal vein thrombosis (PVT), transient elastography

## Abstract

**Background:**

Extrahepatic portal vein obstruction (EHPVO) can be idiopathic or secondary to congenital abnormalities and other conditions in the pediatric population. The severity of the disease can be accurately determined by the precise degree of hepatic fibrosis. Most cases result from portal vein thrombosis (PVT). This study aimed to evaluate the accuracy of liver stiffness measurement (LSM) compared with liver biopsy for assessing liver fibrosis in patients with PVT.

**Methods:**

This retrospective cross-sectional study included children aged 3–18 years with EHPVO/PVT who were referred to the Cairo University Children's Hospital between 1 July 2016 and 31 December 2018. All patients underwent clinical examination, routine laboratory studies, and Doppler ultrasound of the portal system. Fibrosis was staged on liver biopsy using the METAVIR scoring system. Logistic regression was used in an exploratory manner to assess factors associated with hepatic fibrosis.

**Results:**

A total of 20 children were included. Males predominated, accounting for 85%. Splenomegaly was present in 70% of patients and hepatomegaly in 20%. Hepatic fibrosis was absent in 25%, mild in 30%, and moderate in 45% of patients. FibroScan (transient elastography, TE) values were the most strongly associated with fibrosis stage, had good discriminative ability (AUROC 0.912), and appeared to outperform platelet count and liver function tests. Clinical features, including bleeding history, did not correlate with fibrosis stage.

**Conclusion:**

Platelet count and FibroScan (TE) may serve as useful indicators of hepatic fibrosis in children with PVT, with FibroScan (TE) being the strongest associated factor in this sample.

## 1 Introduction

Portal hypertension (PH) is commonly associated with hepatic fibrosis and increases the risk of decompensating events, such as varices, leading to higher morbidity and mortality (1). Although patients with cirrhosis may remain clinically compensated for extended periods, a high hepatic venous pressure gradient (HVPG) is associated with increased liver stiffness and poorer clinical outcomes, highlighting the importance of early assessment and intervention (2).

Extrahepatic portal vein obstruction (EHPVO) represents a major cause of portal hypertension in children, with portal vein thrombosis (PVT) being the most common underlying etiology. Clinically, EHPVO most frequently presents with variceal bleeding. Doppler ultrasonography is the diagnostic modality of choice due to its high sensitivity and specificity (3).

Although portal hypertension is commonly associated with cirrhosis, it can also occur in the absence of cirrhosis due to various etiologies, such as schistosomiasis (4), in a condition known as non-cirrhotic portal hypertension (NCPH) (5) Complications like variceal bleeding, ascites, jaundice, and encephalopathy may still occur even without cirrhosis (6).

The diagnosis of NCPF is typically established by liver biopsy, which may demonstrate features such as obliterative portal venopathy, nodular regenerative hyperplasia, and incomplete septal fibrosis. The presence of these findings in the absence of cirrhosis or significant liver function abnormalities supports the diagnosis (4).

Management of NCPF focuses primarily on preventing and controlling variceal bleeding. Endoscopic therapy is the first-line treatment for acute variceal bleeding, while vasoactive agents may be used temporarily to reduce portal pressure. Additionally, endoscopic screening for gastroesophageal varices at diagnosis and prophylaxis to prevent recurrence is recommended (7).

Liver biopsy has historically been the gold standard for assessing hepatic fibrosis and uses the METAVIR scoring system for staging: F0—no fibrosis; F1—portal fibrosis without septae; F2—portal fibrosis with septae; F3—multiple septae without cirrhosis; and F4—cirrhosis (8–10). Biopsy complications are rare but include bleeding, pain, infection, and occasionally injury to an organ or death (11).

In recent years, non-invasive imaging techniques such as FibroScan (transient elastography, TE), acoustic radiation force impulse (ARFI) imaging, and magnetic resonance elastography (MRE) have gained increasing importance in the assessment of liver fibrosis (12). These modalities offer safer and more practical alternatives to biopsy, particularly in pediatric populations, where minimizing invasive procedures is essential (13, 14).

FibroScan (TE) measures liver stiffness using shear-wave velocities and provides a rapid, reproducible, and non-invasive assessment of hepatic fibrosis. Several pediatric studies have demonstrated a strong correlation between TE measurements and histological fibrosis staging, supporting its role in the evaluation and monitoring of chronic liver diseases in children (15). ARFI imaging is a suitable alternative for patients with obesity or ascites when performed in conjunction with a standard ultrasound device (16), while MRE, although highly accurate, is more time-consuming and costly.

Despite growing evidence supporting the use of TE in pediatric liver disease, data remain limited in children with PVT. A comprehensive understanding of the etiology, clinical presentation, and diagnostic approaches in pediatric portal hypertension is essential for timely intervention and improved outcomes.

In this context, we hypothesized that measuring liver stiffness with TE could provide a reliable, noninvasive method for evaluating hepatic fibrosis in children with PVT. Therefore, this study aimed to assess liver stiffness in pediatric patients with PVT using FibroScan (TE), addressing the current lack of quantitative data in this population.

## 2 Materials and methods

### 2.1 Study design and population

This retrospective cross-sectional study included children aged 3–18 years with extrahepatic portal vein obstruction (EHPVO) secondary to portal vein thrombosis (PVT). Patients were identified from the Hepatology Unit at Cairo University Children's Hospital. The study period spanned from 1 July 2016 to 31 December 2018. Data were collected retrospectively from medical records, including clinical history, laboratory results, imaging findings, FibroScan (transient elastography, TE) measurements, and liver biopsy reports.

### 2.2 Ethics statement

The study protocol was approved by the institutional ethics committee of Cairo University Children's Hospital. All investigations were performed as part of routine clinical care, and written informed consent had been obtained from parents or legal guardians before the procedures. Data were analyzed retrospectively in accordance with institutional ethical guidelines.

### 2.3 Inclusion criteria

Children aged 3–18 years with non-cirrhotic portal hypertension presenting with splenomegaly and/or variceal bleeding were retrospectively identified from medical records. Eligible patients were included consecutively during data extraction if complete clinical, laboratory, Doppler ultrasound, FibroScan (TE) measurements, and liver biopsy data were available.

### 2.3 Exclusion criteria

Children with cirrhosis of known etiology.

Patients with obesity or massive ascites were excluded.

Patients with chronic liver diseases: chronic viral hepatitis B/C, autoimmune hepatitis, and primary biliary cirrhosis.

### 2.4 Bias statement

To minimize selection bias, only patients with complete clinical, laboratory, imaging, and histopathology records were included.

### 2.5 Sampling

All eligible patients who met the inclusion criteria during the study period; therefore, no formal sample size calculation was performed.

### 2.6 Methods

A) Clinical Assessment

A detailed clinical history was taken, and a physical examination was performed for each child using a standardized liver disease assessment sheet.

B) Laboratory Investigations

Complete blood count (CBC).

Liver function tests: alanine transaminase (ALT), aspartate transaminase (AST), alkaline phosphatase (ALP), total bilirubin, serum albumin.

Coagulation profile: activated partial thromboplastin time (APTT), Prothrombin Time (PT), and International Normalized Ratio (INR).

C) Doppler Ultrasound

Assessment of the portal vein and detection of vascular abnormalities.

D) Liver Biopsy

Biopsies were performed percutaneously after obtaining written informed consent and evaluating bleeding risk before the procedure. Under sedation, samples were obtained using intravenous (IV) midazolam with ultrasound guidance to select an appropriate site in the right hepatic lobe. Specimens measuring at least 1.5 cm were formalin-fixed and stained with hematoxylin-eosin, reticulin, and Masson trichrome.

Histological assessment was conducted by an experienced pathologist blinded to all clinical and FibroScan (TE) measurements data. A minimum of 10 portal tracts was required for adequate evaluation. Fibrosis and necroinflammatory activity were staged according to the METAVIR scoring system (F0–F4).

For analytical purposes, fibrosis was categorized as absent (F0) or present (F1–F2) to enable statistical comparison within the study sample.

Due to the retrospective design, the exact time interval between transient elastography and liver biopsy was not consistently documented. However, both assessments were performed as part of routine clinical evaluation within the same period of patient management, and no major clinical events were recorded between the two procedures.

E) FibroScan (TE; liver stiffness measurement, LSM). Transient elastography was performed using a FibroScan device (Echosens, Paris, France). The specific device model was not recorded; however, all measurements were obtained using standard vibration-controlled transient elastography (VCTE) technology. The examination was performed in the dorsal decubitus position, with the right arm maximally abducted to widen the intercostal space. The probe was placed over the right lobe of the liver using coupling gel, avoiding large vascular structures. A total of 10 valid measurements were obtained for each patient. The median liver stiffness measurement (LSM, kPa) was recorded as the final value, with an interquartile range and a success rate of ≥60% considered acceptable for analysis. Measurements were performed at a depth of 2.5–5.5 cm for children under 7 years of age and 2.5–6.5 cm for older children (17, 18).

### 2.7 Statistical analysis

Data were coded and analyzed using SPSS version 17. Descriptive statistics included mean ± SD, median, and interquartile range (IQR), and frequencies (%). For analytical tests, the following were used: (1) ANOVA with *post-hoc* Tukey, (2) Kruskal–Wallis for non-parametric comparisons, (3) chi-square for categorical data, and (4) Spearman correlation for relationships between variables. Potential confounding variables were assessed using multivariable logistic regression. Logistic regression was used in an exploratory manner to identify and assess factors associated with hepatic fibrosis, and ROC curves were used to evaluate model performance. Statistical significance was defined as a *P*-value <0.05. Patients with missing key variables (FibroScan, Doppler, or biopsy data) were excluded at the eligibility stage. All statistical tests were two-tailed.

## 3 Results

### 3.1 Baseline clinical, demographic, and laboratory characteristics

During the study period, 32 children with extrahepatic portal vein obstruction (EHPVO) secondary to portal vein thrombosis (PVT) were screened for eligibility. Twelve patients were excluded due to incomplete clinical, laboratory, imaging, or histopathological data, leaving 20 patients with complete datasets for final analysis.

The study population had a mean age of 8.5 ± 3.7 years with marked male predominance. Splenomegaly was the most frequent clinical finding, whereas hepatomegaly was less common, and no patient presented with ascites. A history of gastrointestinal bleeding and prior blood transfusion was documented in a substantial proportion of cases. Detailed demographic and clinical characteristics are summarized in Table 1, while laboratory findings are provided in Supplementary Table S1.

**Table 1:** Baseline demographic characteristics, clinical presentation, and physical examination findings of pediatric patients with portal vein thrombosis (*n* = 20).

Age(yrs) (mean ± SD)		8.50	±3.71
Sex	Male	17	85.0%
	Female	3	15.0%
Bleeding	None	9	45.0%
	Hematemesis	3	15.0%
	Melena	2	10.0%
	Hematemesis &Melena	5	25.0%
	Hematemesis, melena & bleeding\ rectum	1	5.0%
Similar attack	Negative	17	85.0%
	Positive	3	15.0%
Blood transfusion	Negative	4	20.0%
	Positive	16	80.0%
Similar condition	Negative	18	90.0%
	Positive	2	10.0%
Consanguinity	Negative	14	70.0%
	Positive	6	30.0%
WT(kg) (mean ± SD)		24.08	±8.86
HT(cm) (mean ± SD)		118.50	±22.68
Splenomegaly	Negative	6	30.0%
	Positive	14	70.0%
Hepatomegaly	Negative	16	80.0%
	Positive	4	20.0%
Ascites	Negative	20	100.0%
	Positive	0	0.0%

Data expressed as mean ± SD or as frequency (Number-percent).

SD, standard deviation.

### 3.2 Histopathological and Doppler findings

Histopathological assessment showed that hepatic fibrosis was absent in 25% of patients, mild in 30%, and moderate in 45% of patients. Details are provided in Table 2. No patients demonstrated advanced fibrosis (F3–F4). Additional Doppler ultrasound findings are provided in Supplementary Table S2.

**Table 2:** Distribution of hepatic fibrosis stages and necroinflammatory activity grades according to the METAVIR scoring system in the study population (*n* = 20).

Variable	Category	Number (*n*)	Percentage (%)
Fibrotic stages	F0	5	25.0%
	F1	6	30.0%
	F2	9	45.0%
necroinflammatory activity	A0	11	55.0%
	A1	5	25.0%
	A2	4	20.0%

METAVIR = scoring system for fibrosis (F0–F4) and necroinflammatory activity (A0–A3).

### 3.3 Associations between fibrosis stage and clinical, laboratory, and imaging parameters

No significant association was observed between fibrosis stages and clinical characteristics (Supplementary Table S3). However, significant associations were identified between fibrosis stages and selected laboratory parameters, as shown in Table 3. The relationship between fibrosis stage and necroinflammatory activity is presented in Supplementary Table S4.

**Table 3:** Comparison of laboratory parameters across hepatic fibrosis stages (F0–F2) in pediatric patients with portal vein thrombosis.

Laboratory parameter	F0 (mean ± SD or median [IQR])		F1 (mean ± SD or median [IQR])		F2 (mean ± SD or median [IQR])		*P*-value
HB	10.95	±.68	11.80	±1.08	10.80	±1.34	0.25
RBC'S	4.41	±.38	4.78	±.67	4.41	±.41	0.34
WBC'S	5.27	±3.72	6.75	±1.62	5.74	±1.59	0.55
PLT	87.12	±54.00	241.17	±166.87	140.49	±66.26	0.07
ALP	200.40	±34.28	222.00	±59.27	199.11	±51.96	0.67
TSB	1.10	±.42	1.30	±1.96	1.60	±2.50	0.9
Sr.Albumin	3.60	±1.58	4.02	±.74	4.51	±.43	0.2
PT (\sec)	12.42	±.11	13.72	±1.33	12.72	±.67	0.046*
INR	1.04	±.09	1.19	±.09	1.04	±.10	0.02*
APTT(\sec)	35.20	±4.76	37.17	±7.11	38.76	±7.48	0.65
SGPT	18.00	14.00–34.00	22.50	18.00–35.00	22.00	19.00–31.00	0.77
SGOT	31.00	30.00–36.00	32.50	31.00–35.00	36.00	33.00–51.00	0.3

HB, hemoglobin; g/dL, grams per deciliter; RBCs, red blood cells; 10⁶/µL, million cells per microliter; WBCs, white blood cells; 10^3^/µL, thousand cells per microliter; PLT, platelets; ALP, alkaline phosphatase; U/L, units per liter; TSB, total serum bilirubin; mg/dL, milligrams per deciliter; PT, prothrombin time; s, seconds; INR, international normalized ratio; APTT, activated partial thromboplastin time; ALT, alanine aminotransferase; AST, aspartate aminotransferase; SD, standard deviation; P, probability.

**Figure 1:**
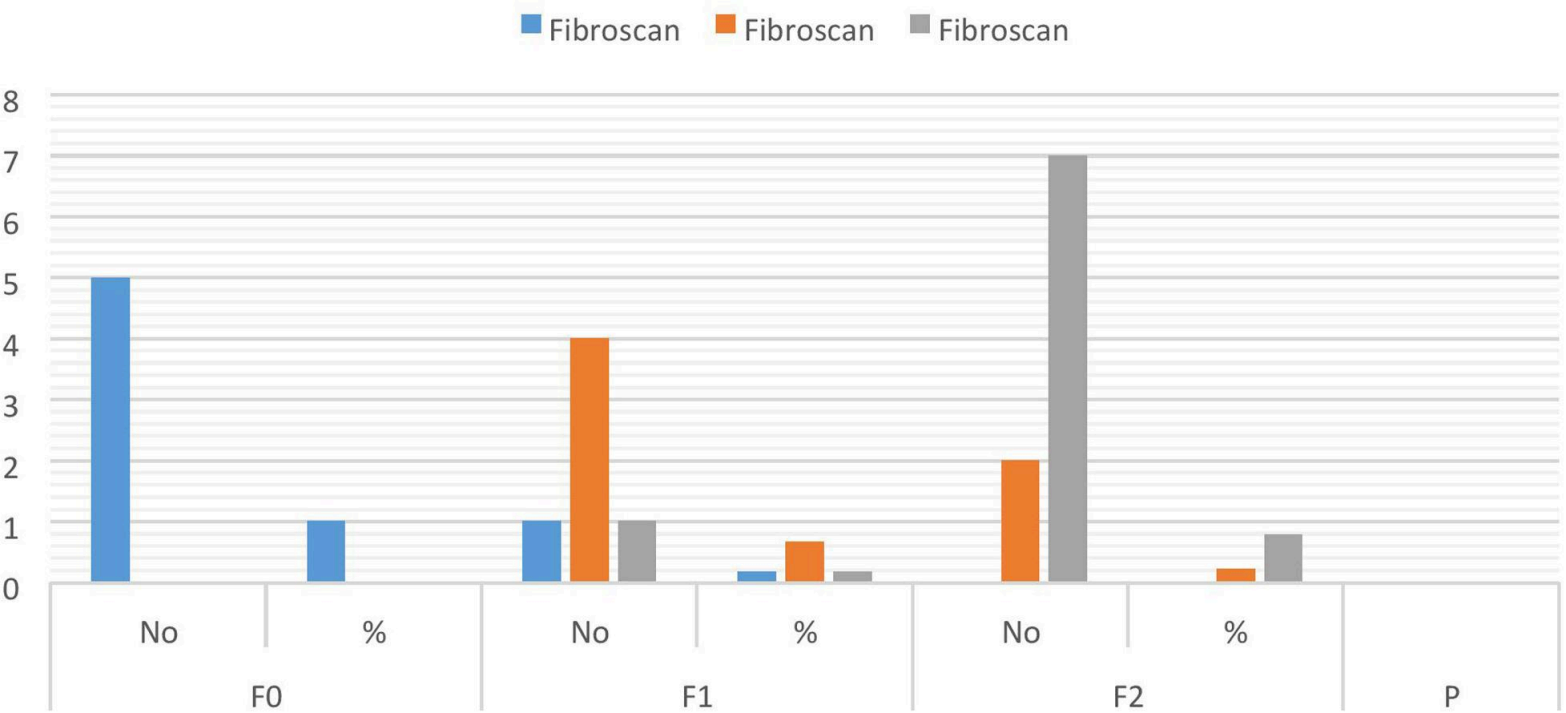
Distribution of liver stiffness measurements (FibroScan values) across hepatic fibrosis stages (METAVIR F0–F2). Alt text: Comparative presentation of fibrotic stages and corresponding grades of liver stiffness measured by FibroScan.

**Figure 2:**
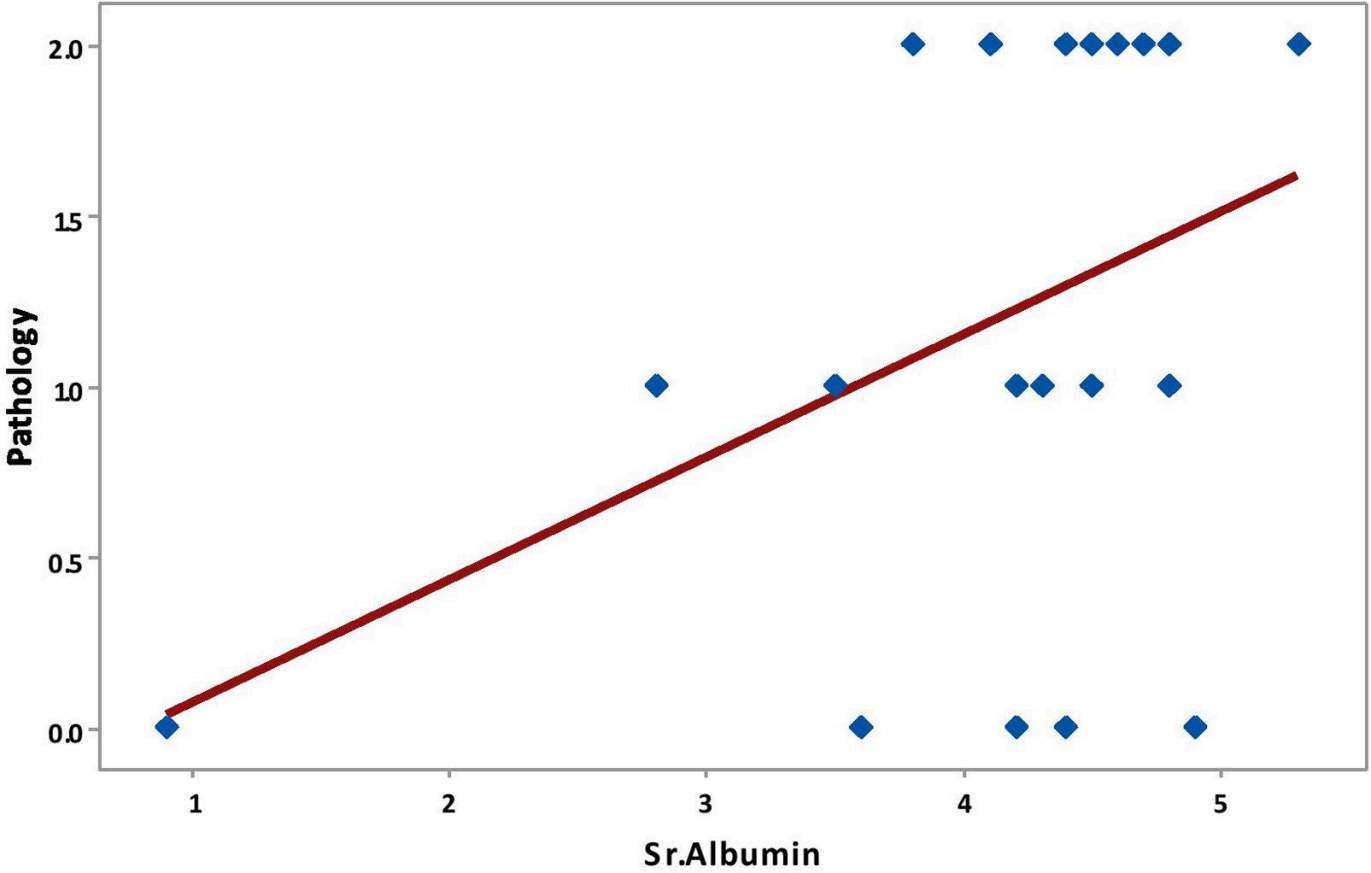
Correlation between hepatic fibrosis stage (METAVIR F0–F2) and liver stiffness measurements obtained by FibroScan. Alt text: Association between hepatic pathology and liver stiffness values measured by FibroScan.

Analysis of PVT grades did not demonstrate a consistent association with fibrosis stages (Supplementary Table S5). In contrast, FibroScan (transient elastography, TE) measurements showed a strong and statistically significant association with fibrosis stages (*P* < 0.001), as presented in Table 4, Figures 1, 2.

**Table 4:** Association between liver stiffness measurements (fibroScan grades) and histologically confirmed hepatic fibrosis stages (METAVIR F0–F2) in the study cohort.

FibroScan grade		F0 *n* (%)		F1 *n* (%)		F2 *n* (%)		*P*-value
Fibroscan (KPscal)	F0	5	100.0%	1	16.7%	0	0.0%	<0.001**
	F1	0	0.0%	4	66.7%	2	22.2%	
	F2	0	0.0%	1	16.7%	7	77.8%	

kPa, kilopascal; F0–F2 = METAVIR fibrosis stages; P:Probability.

** High significance Test used: Chi-square.

The correlation analysis between fibrosis stage, FibroScan (TE), and clinical and laboratory parameters is summarized in Supplementary Table S6.

### 3.4 Regression analysis and diagnostic performance of FibroScan

Univariate logistic regression analysis of factors associated with hepatic fibrosis is provided in Supplementary Table S7. Multivariate logistic regression analysis identified FibroScan (TE) as the strongest factor associated with hepatic fibrosis in this cohort (Table 5).

**Table 5:** Multivariable logistic regression analysis identifying factors associated with the presence of hepatic fibrosis (F1–F2 vs. F0) in pediatric patients with portal vein thrombosis .

Variable	*P*-value	OR	95%CI
Fibroscan	.045*	7.407	1.047–52.386

OR, odd's ratio CI, confidence interval *:mild significance <0.05 **:moderate significance <0.01 ***:high significance<0.001.

**Figure 3:**
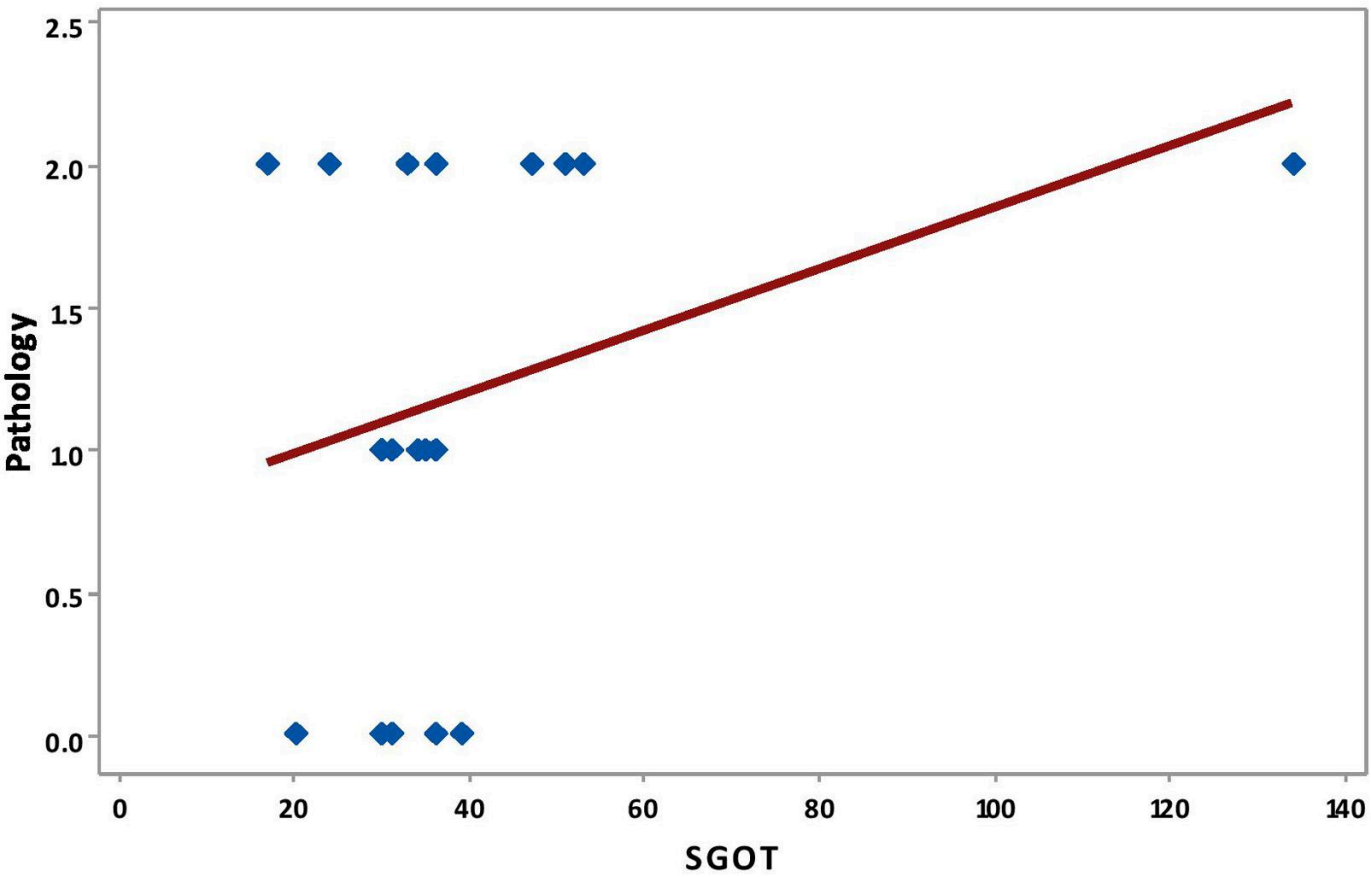
Receiver operating characteristic (ROC) curve evaluating the accuracy of FibroScan for detecting hepatic fibrosis. Alt text: Receiver operating characteristic curve showing the performance of FibroScan in identifying hepatic fibrosis in patients with portal vein thrombosis.

Receiver operating characteristic (ROC) curve analysis demonstrated good discriminative performance of FibroScan for identifying hepatic fibrosis, with an area under the curve (AUC) of 0.912 (Table 6, Figure 3).

**Table 6:** Receiver operating characteristic (ROC) curve analysis demonstrating the diagnostic performance of fibroScan for detecting hepatic fibrosis in pediatric portal vein thrombosis .

AUC	P	95% CI
.912	.003*	.776–1.000

AUC, area under the curve; P, probability; CI, confidence interval.

* Significance<0.05.

**Figure 4:**
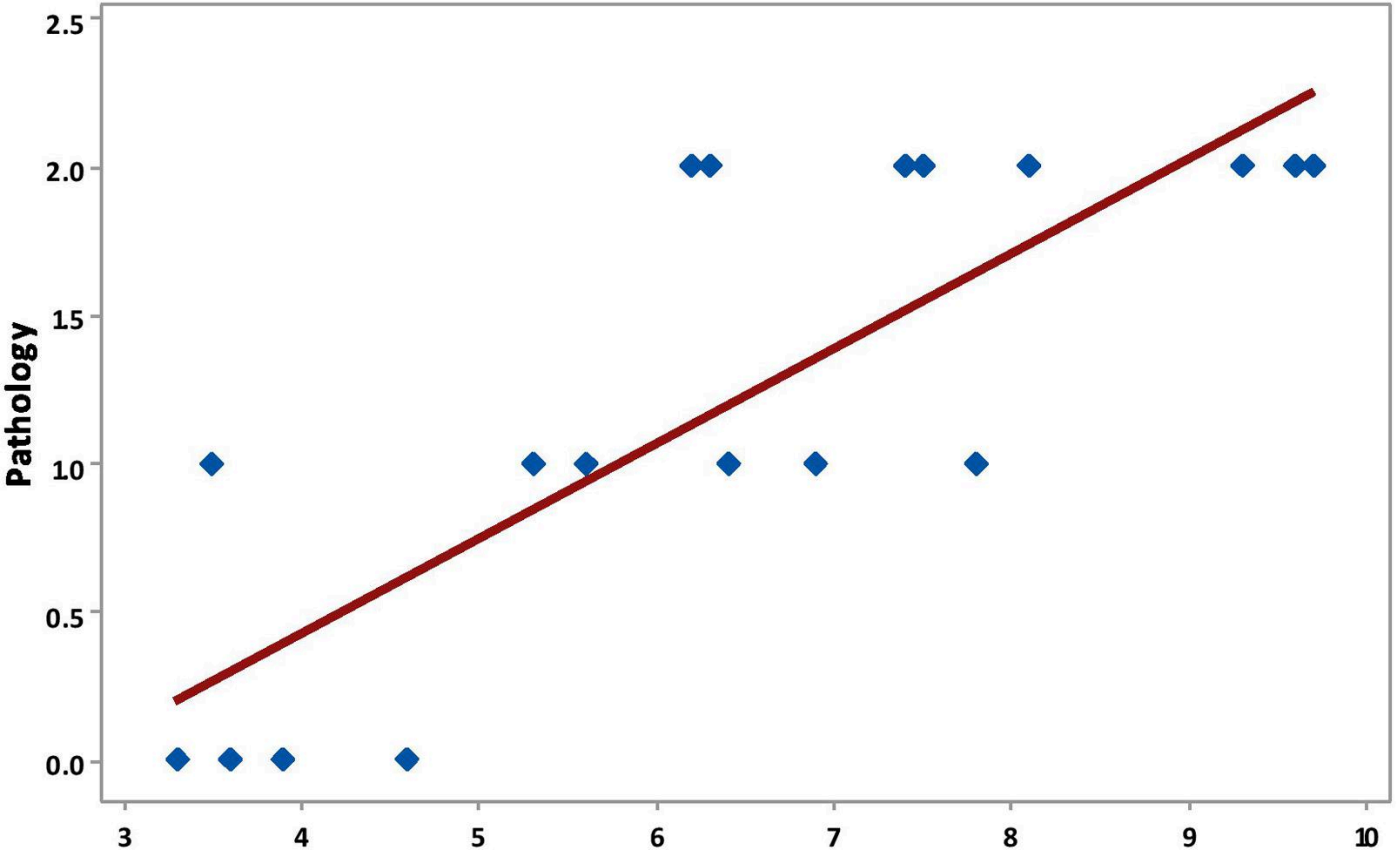
Association between hepatic fibrosis stage (METAVIR F0–F2) and serum albumin levels. Alt text: Relationship between hepatic pathology and serum albumin levels in the studied patients.

**Figure 5:**
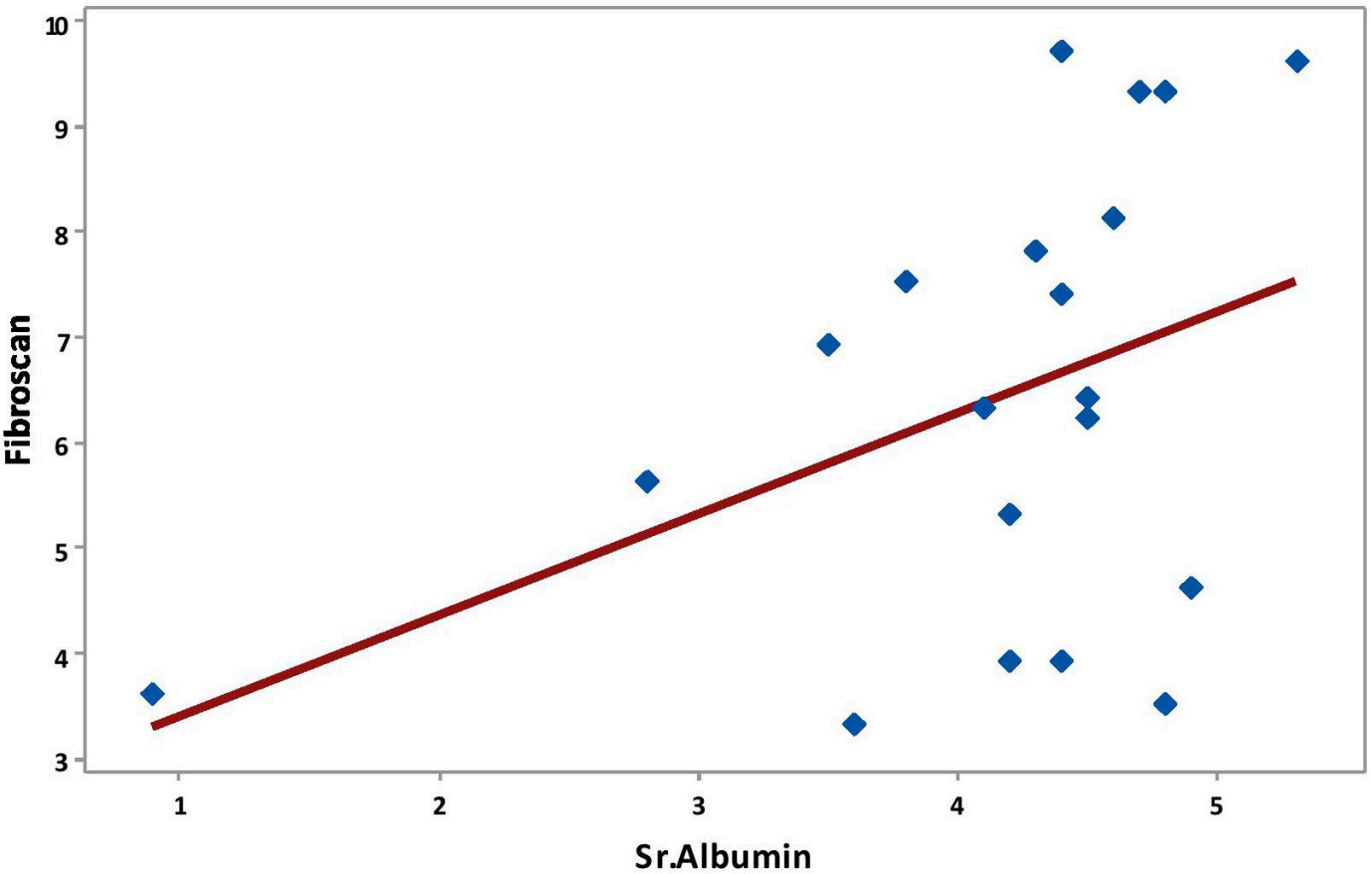
Association between hepatic fibrosis stage (METAVIR F0–F2) and aspartate aminotransferase (AST) levels. Alt text: Relationship between hepatic pathology and aspartate aminotransferase (SGOT) levels.

**Figure 6:**
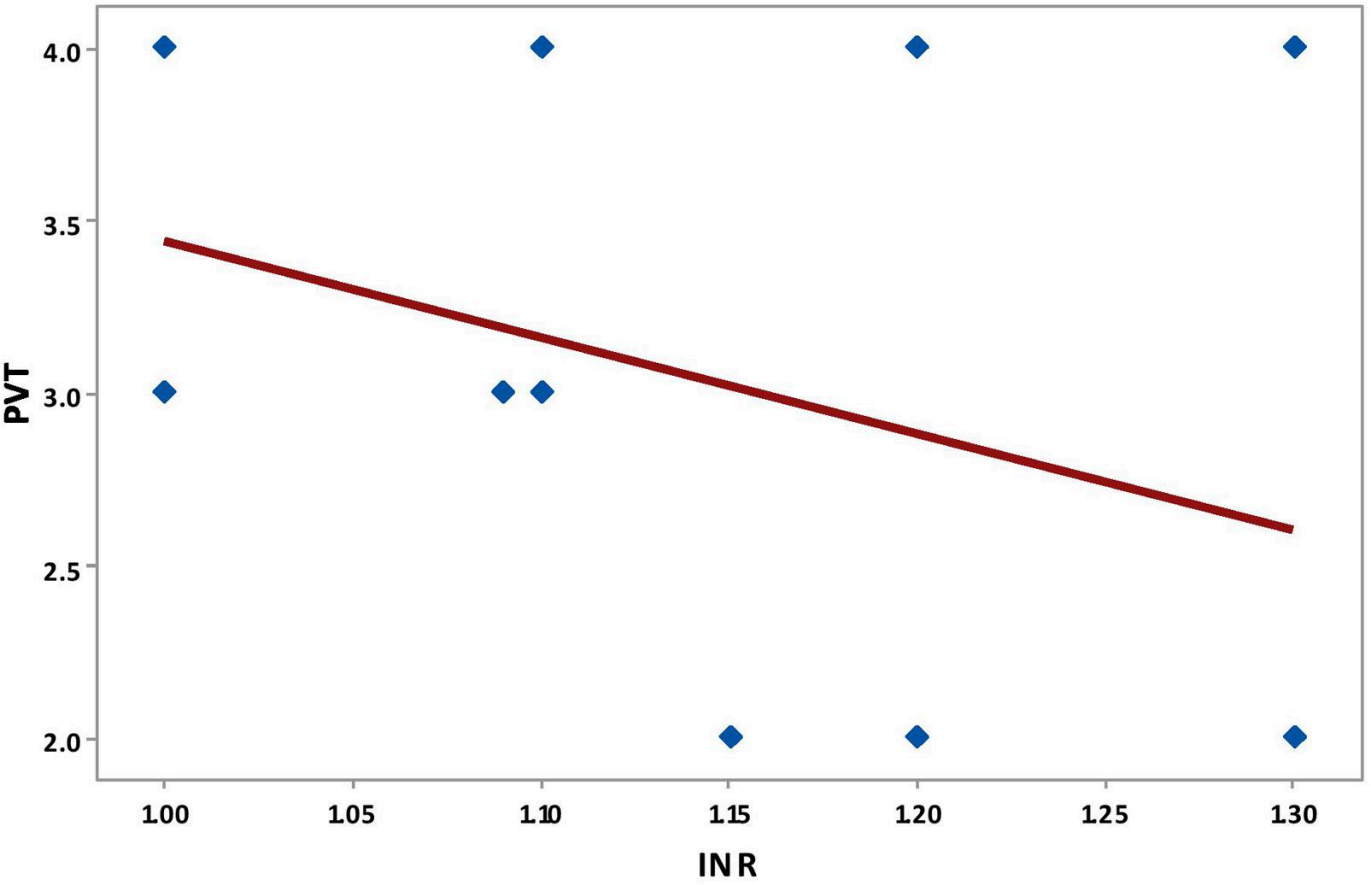
Association between liver stiffness measurements (FibroScan) and serum albumin levels. Alt text: Relationship between liver stiffness measured by FibroScan and serum albumin levels.

**Figure 7:**
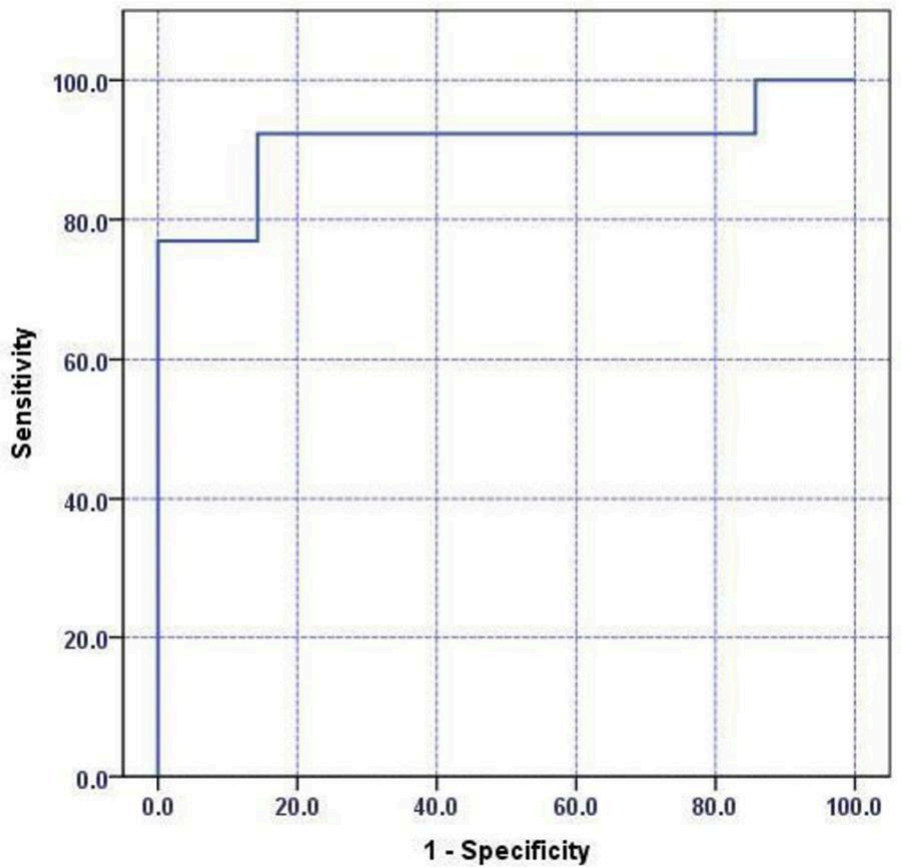
Association between portal vein thrombosis grade and international normalized ratio (INR). Alt text: Relationship between grades of portal vein thrombosis and international normalized ratio values.

Additional relationships between pathological findings, FibroScan (TE) measurements, and laboratory parameters are illustrated in Figures 4–7.

## 4 Discussion

Our findings suggest that FibroScan (transient elastography, TE) correlates with fibrosis assessment in pediatric patients with portal vein thrombosis (PVT). These findings are consistent with recent studies demonstrating that FibroScan (TE) is a reliable and reproducible non-invasive tool for assessing liver fibrosis in children, with good correlation with histological staging and clinical severity (19, 20). Recent pediatric evidence further supports its role in both diagnosis and longitudinal monitoring, potentially reducing the need for invasive liver biopsy in selected cases (15).

A study by Sharma et al. (21) evaluated liver stiffness (LS) using FibroScan (TE) in adults with extrahepatic portal vein obstruction (EHPVO). In contrast, our study focused on children with PVT. We found no significant correlation between FibroScan (TE)-derived liver stiffness measurements and bleeding, ALP, AST, or ALT, unlike Sharma et al. (21) who reported significantly higher LS in patients with bleeds. This discrepancy may be due to the smaller number of pediatric patients with PVT in our cohort. Additionally, Sharma et al. (21) observed that children with PVT and variceal bleeding had lower LS values than patients with cirrhosis, using a cutoff of 5.9 kPa, which is generally consistent with our findings regarding fibrosis scores. This difference may also reflect the heterogeneity between adult and pediatric populations, as liver stiffness values in children can be influenced by age, underlying disease etiology, and developmental factors, making direct comparison with adult cohorts challenging (20).

In our study, liver biopsy showed a significant association (*P* = 0.036) between hepatic fibrosis stage and necroinflammatory activity, in contrast to findings reported by Dyvorne et al. (22) likely due to differences in liver disease aetiologies. Fibrotic stages did not correlate with ALT, ALP, or platelet count (PLT). However, they showed a highly significant association with FibroScan (TE), in agreement with Corpechot et al. (23) who reported that FibroScan (TE) outperformed biochemical markers in identifying significant fibrosis or cirrhosis.

Doppler ultrasound did not show a significant correlation between PVT grades and FibroScan (TE)-derived liver stiffness measurements, in contrast to Sharma et al. (21) but aligns with Madhusudhan et al. (24) reflecting differences in stiffness measurement techniques and the absence of a healthy control comparison in our study. This observation is supported by recent evidence indicating that liver stiffness measurements primarily reflect parenchymal fibrosis rather than vascular severity, which may explain the lack of direct correlation with PVT grading (15).

Our findings suggest that FibroScan (TE) correlates strongly with fibrosis stages (*P* < 0.001), consistent with De Lédinghen et al. (17), Sandrin et al. (18), Bota et al. (25) and Dyvorne et al. (22) who demonstrated strong agreement between noninvasive measures and histological fibrosis. Logistic regression and ROC analysis further indicated that FibroScan (TE) was the most strongly associated factor in this sample for hepatic fibrosis in children with PVT (AUROC 0.912), consistent with Nobili et al. (26) and Cardoso et al. (27). Recent advances in imaging have further strengthened the role of elastography-based techniques in pediatric hepatology. Modalities such as transient elastography, acoustic radiation force impulse imaging, and magnetic resonance elastography have demonstrated high diagnostic accuracy for liver fibrosis across various pediatric liver diseases. However, their application in specific conditions remains less well established, particularly in pediatric populations (14).

Overall, our findings suggest that FibroScan (TE) may be a useful non-invasive tool for assessing hepatic fibrosis in pediatric patients with PVT, with potential to inform clinical management and reduce the need for invasive biopsy. However, the absence of advanced fibrosis stages (F3–F4) in our cohort limits the evaluation of FibroScan (TE) performance in identifying severe fibrosis or cirrhosis. These findings should be interpreted with caution and validated in larger prospective cohorts before being generalized to broader pediatric populations.

The present study has several limitations. First, the sample size was relatively small (*n* = 20), which limited statistical power and may have affected the reliability of multivariable analyses. In particular, the use of multivariable logistic regression with a limited number of events increases the risk of overfitting, potentially leading to unstable effect estimates. The wide confidence intervals observed in the regression model further reflect the limited sample size and reduced statistical precision of the estimates. Additionally, the recommended events-per-variable ratio for logistic regression may not have been met, thereby reducing the model's robustness.

Second, the retrospective design relied on the accuracy and completeness of pre-existing medical records, which may have introduced information bias. Furthermore, the lack of precise documentation of the time interval between transient elastography and liver biopsy may have affected the comparability between liver stiffness measurements and histological findings.

Third, no patients in our cohort had advanced fibrosis (F3–F4). This restricted fibrosis spectrum limits the ability to assess FibroScan (TE)'s diagnostic performance in detecting severe fibrosis or cirrhosis. It may reduce the generalizability of our findings to patients with more advanced liver disease. Therefore, our results should be interpreted primarily in the context of early-to-moderate fibrosis in pediatric PVT.

Finally, the study population was derived from a single tertiary care center, which may limit the external validity of the findings.

## 5 Conclusion

In this small retrospective cohort, liver stiffness measurement using FibroScan (transient elastography, TE) was associated with biopsy-assessed fibrosis in children with portal vein thrombosis (PVT). These findings suggest that transient elastography may have a role as a non-invasive tool for assessing liver fibrosis in this population. However, given the limited sample size and study design, the results should be interpreted with caution and require validation in larger, prospective studies before wider clinical application.

## Data Availability

The original contributions presented in the study are included in the article/Supplementary Material, further inquiries can be directed to the corresponding author/s.
