# Supplementary figures and images for: Liver stiffness measured by transient elastography is associated with hepatic Fibrosis in children with portal vein thrombosis: a retrospective cross-sectional study
Source: Front Pediatr. 2026 Jun 5;14:1779390. doi: 10.3389/fped.2026.1779390 (PMC13279521; doi:10.3389/fped.2026.1779390)

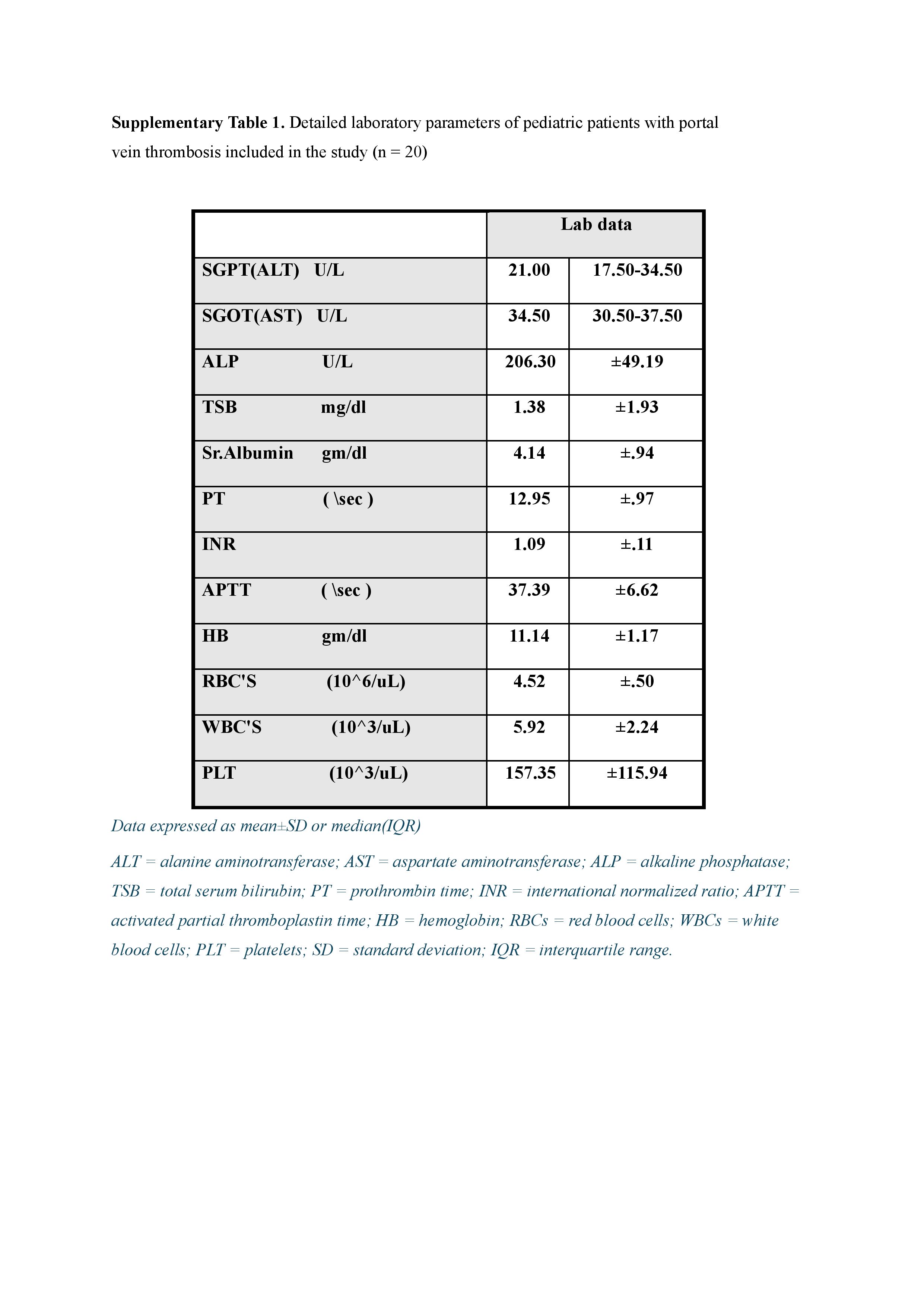

Supplement: Supplementary file 1 [file Image1.jpeg]

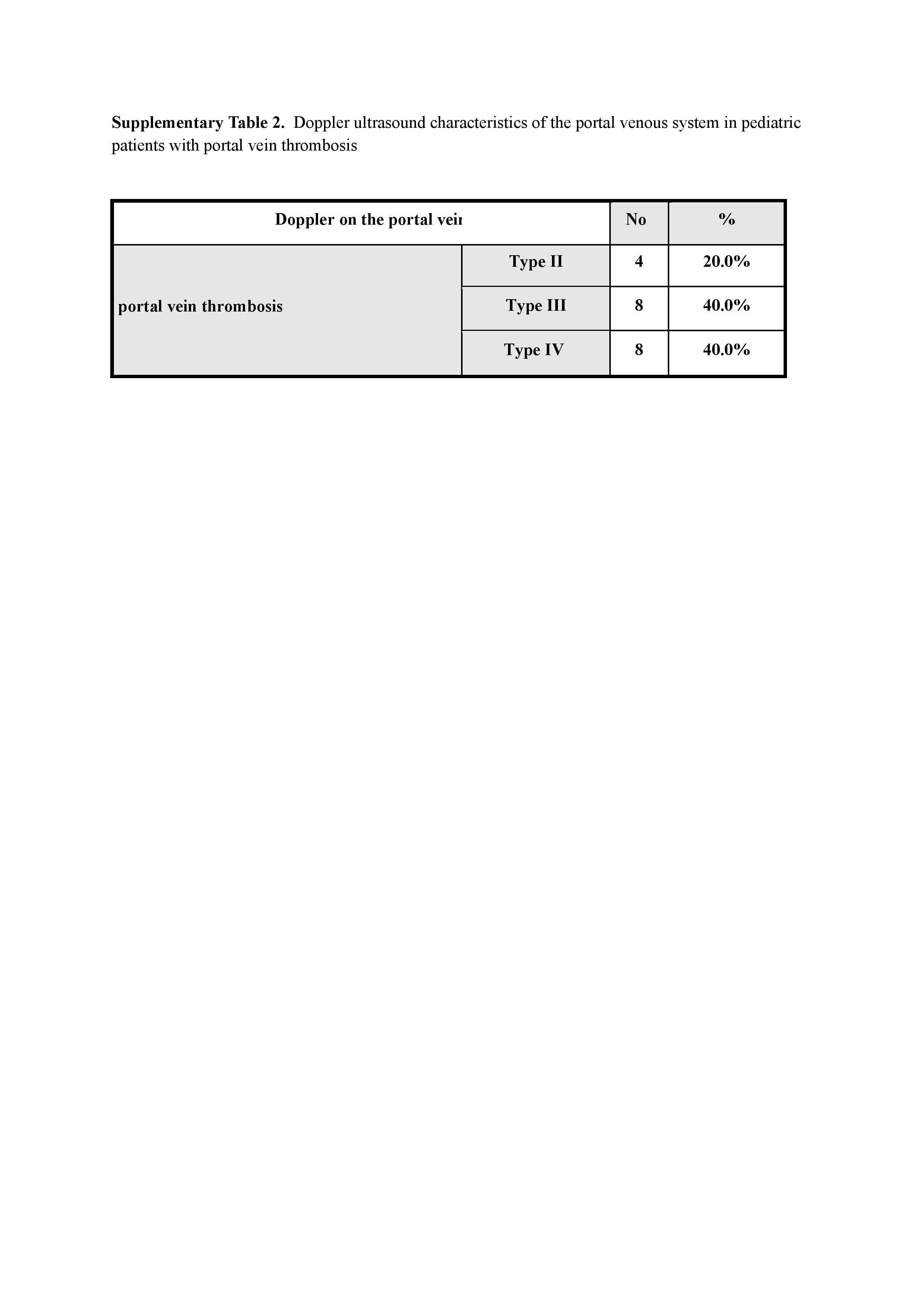

Supplement: Supplementary file 2 [file Image2.jpeg]

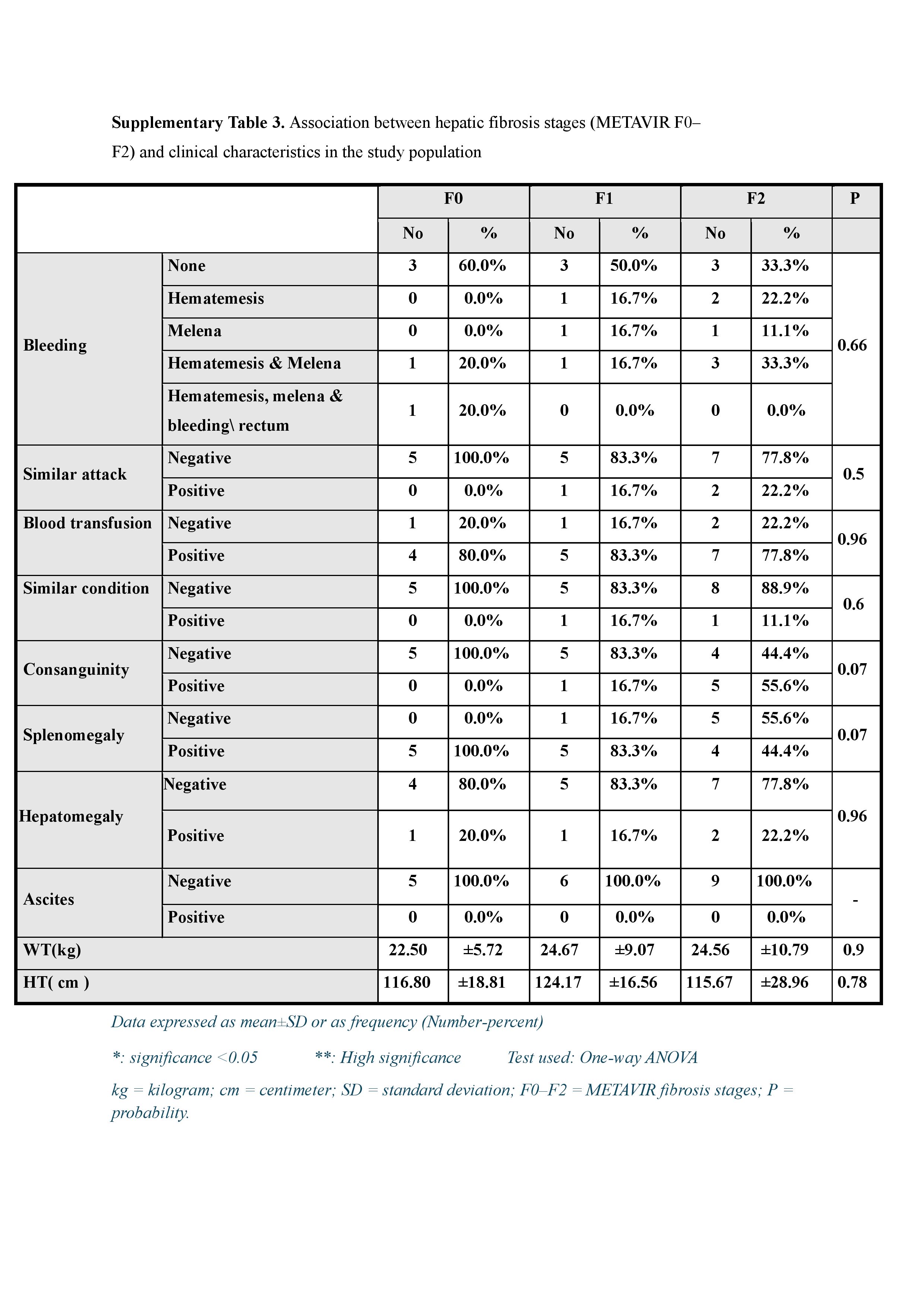

Supplement: Supplementary file 3 [file Image3.jpeg]

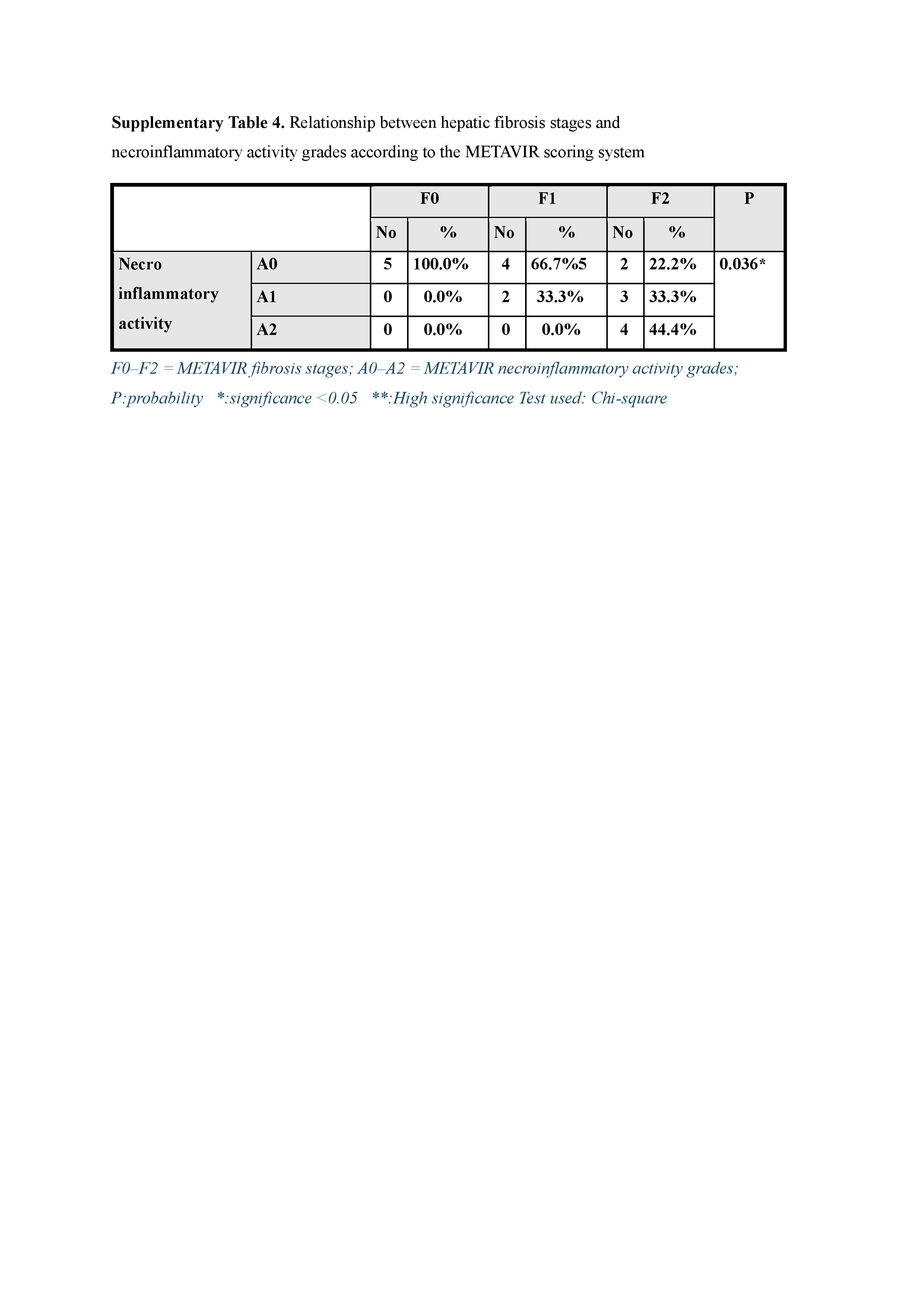

Supplement: Supplementary file 4 [file Image4.jpeg]

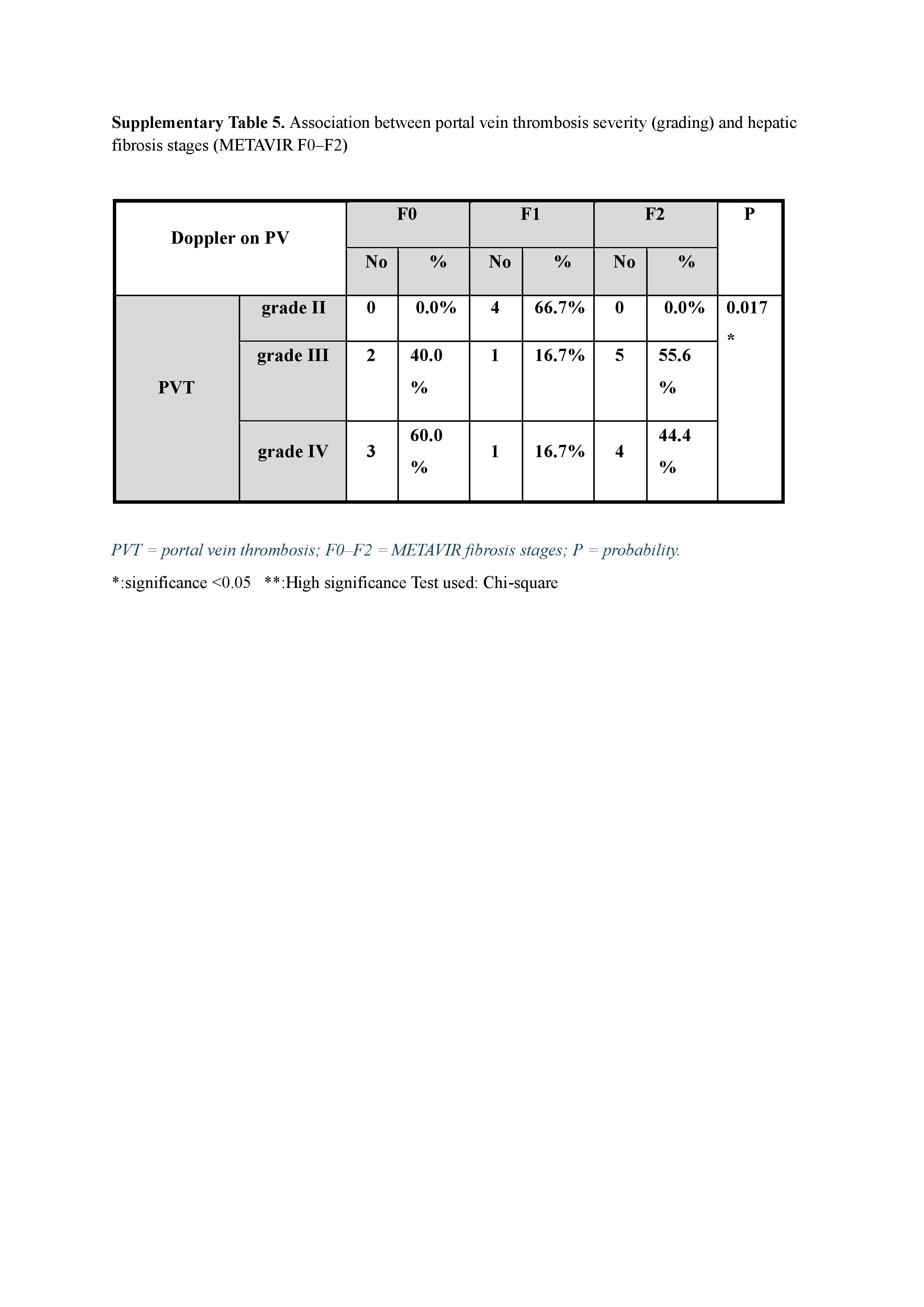

Supplement: Supplementary file 5 [file Image5.jpeg]

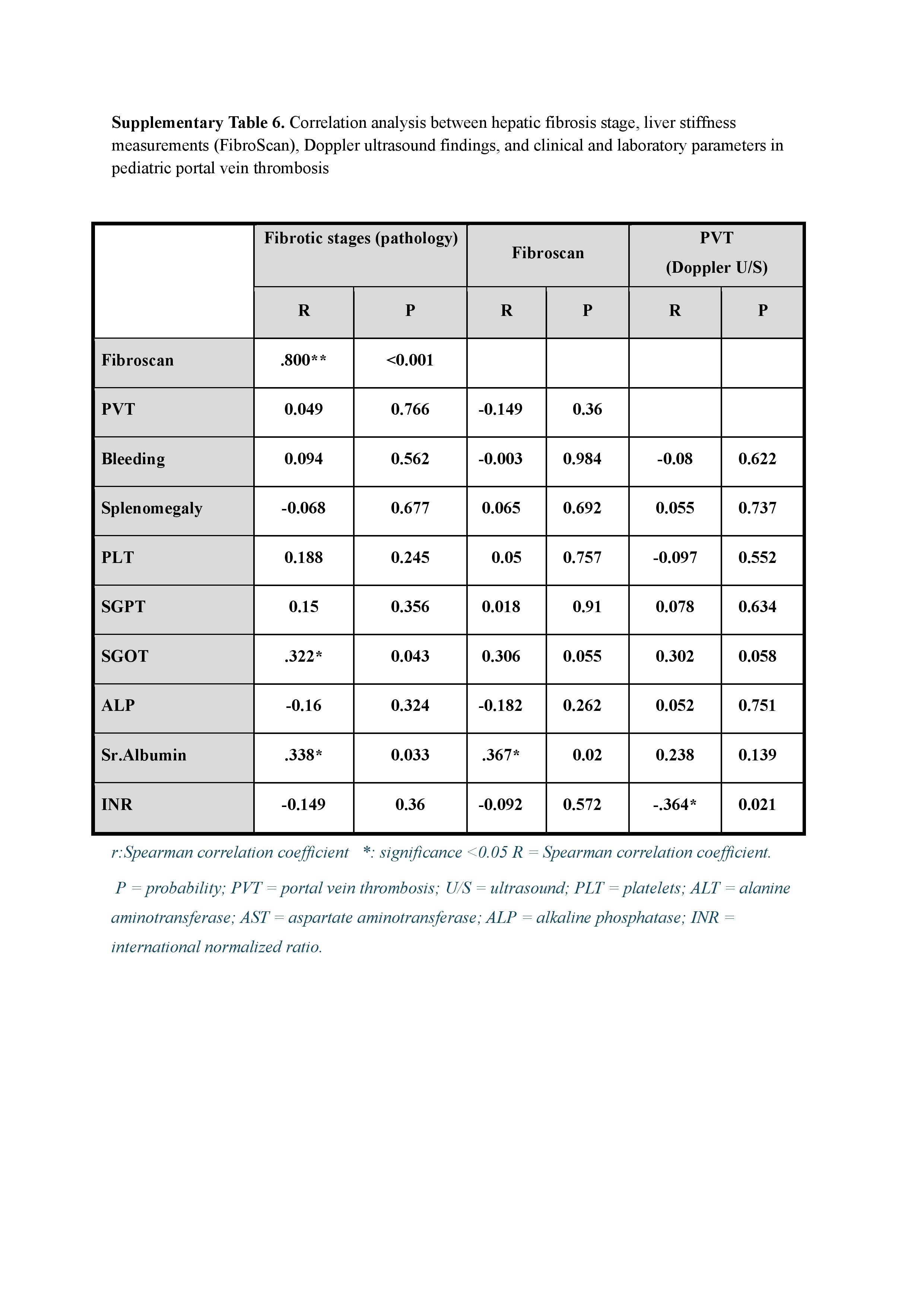

Supplement: Supplementary file 6 [file Image6.jpeg]

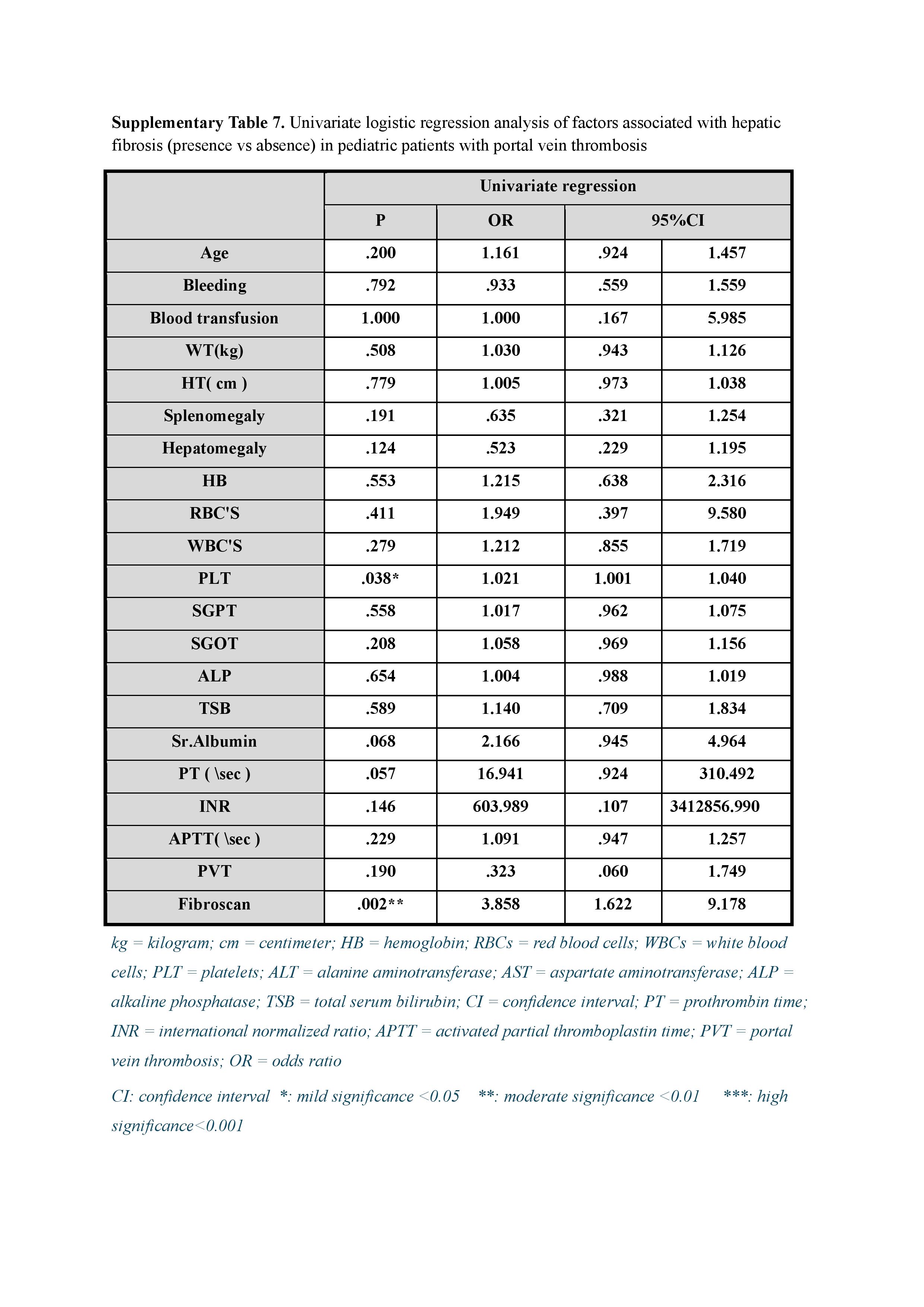

Supplement: Supplementary file 7 [file Image7.jpeg]
